# Synthesis and crystal structure analysis of bis­(benzo­thia­zole-2-thiol­ato-κ*S*)(1,10-phen­anthroline-κ^2^*N*,*N*′)zinc(II)

**DOI:** 10.1107/S2056989025005468

**Published:** 2025-06-24

**Authors:** Gulchekhra Abdullayeva, Batirbay Torambetov, Shakhnoza Kadirova, Shahlo Daminova

**Affiliations:** a National University of Uzbekistan named after Mirzo Ulugbek 4 University St Tashkent 100174 Uzbekistan; b Physical and Material Chemistry Division CSIR-National Chemical Laboratory,Pune 411008 India; c Uzbekistan–Japan Innovation Center of Youth, University Street 2B, Tashkent, 100095, Uzbekistan; University of Hyogo, Japan

**Keywords:** crystal structure, mol­ecular structure, zinc complex, 2-mercaptobenzo­thia­zole, 1,10-phenanthroline, Hirshfeld surface

## Abstract

The coordination complex [Zn(C_7_H_4_NS_2_)_2_(C_12_H_8_N_2_)] or [Zn(MBT)_2_(phen)], was synthesized using ethanol solutions of Zn(CH_3_COO)_2_·2H_2_O, 1,10-phenanthroline (phen) and 2-mercaptobenzo­thia­zole (MBTH-neutral). Single-crystal X-ray diffraction analysis revealed that the zinc atom resides on a crystallographic twofold axis within the asymmetric unit. In the complex, the zinc atom coordinates two 2-mercaptobenzo­thia­zolate (MBT; anionic form of MBTH) ligands in a monodentate fashion through their sulfur atoms, while phenanthroline acts as a bidentate ligand, chelating the zinc center. Further structural analysis, including Hirshfeld surface and two-dimensional fingerprint plot studies, indicated the presence of multiple inter­molecular inter­actions, particularly C—H⋯N and C—H⋯π interactions, contributing to the cohesion and packing of the crystal structure.

## Abstract 2

The coordination complex [Zn(phen)(MBT)_2_], which exhibits a distorted tetra­hedral geometry, was synthesized. Single-crystal X-ray analysis and Hirshfeld surface analysis revealed the presence of several inter­molecular C—H⋯N and C—H⋯π inter­actions.

## 1. Chemical context

2-Mercaptobenzo­thia­zole (MBTH) is a heterocyclic aromatic derivative of benzo­thia­zole, containing a fused benzene and thia­zole ring. The structural modification significantly alters the chemical properties of the mol­ecule, making MBTH more suitable for industrial applications, particularly as a vulcanization accelerator in the rubber industry (Pattanasiriwisawa *et al.*, 2008 ▸). MBTH can exist in two tautomeric forms *i.e.* thiol and thione due to the presence of the mercapto (–SH) or thione (=S) functional group within the benzo­thia­zole rings (Yekeler & Yekeler, 2006 ▸; Castro *et al.*, 1993 ▸; Rakhmonova *et al.*, 2022 ▸). This tautomerism allows for flexibility in coordination behavior, making it a versatile ligand in coordination chemistry, capable of forming stable complexes with metal atoms *via* the exocyclic sulfur atom (Jeannin *et al.*, 1979 ▸; Bravo *et al.*, 1985 ▸), or through its nitro­gen atom (Dey *et al.*, 2011 ▸; Li *et al.*, 2013 ▸), and of acting as a chelating ligand depending on the tautomeric state and the coordination environment. This differential binding ability of MBTH derivatives enhances its applicability in the synthesis of metal complexes for optical properties (Dey *et al.*, 2011 ▸) and has applications in electropolymerization (de Fátima Brito Sousa *et al.*, 1997 ▸). The present works describes the molecular and crystal structure of the zinc complex [Zn(MBT)_2_(phen)]; in addition to MBTH, 1,10-phenanthroline (phen) was used as a co-ligand for its rigid, planar, bidentate nature, and strong metal-chelating ability, and π-accepting properties. The presence of planar conjugated rings enhances complex stability, supports supra­molecular inter­actions (π–π stacking, C—H⋯π), and improves both the structural and functional aspects of the coordination complex (Bencini *et al.*, 2010 ▸; Chaurasia *et al.*, 2021 ▸).
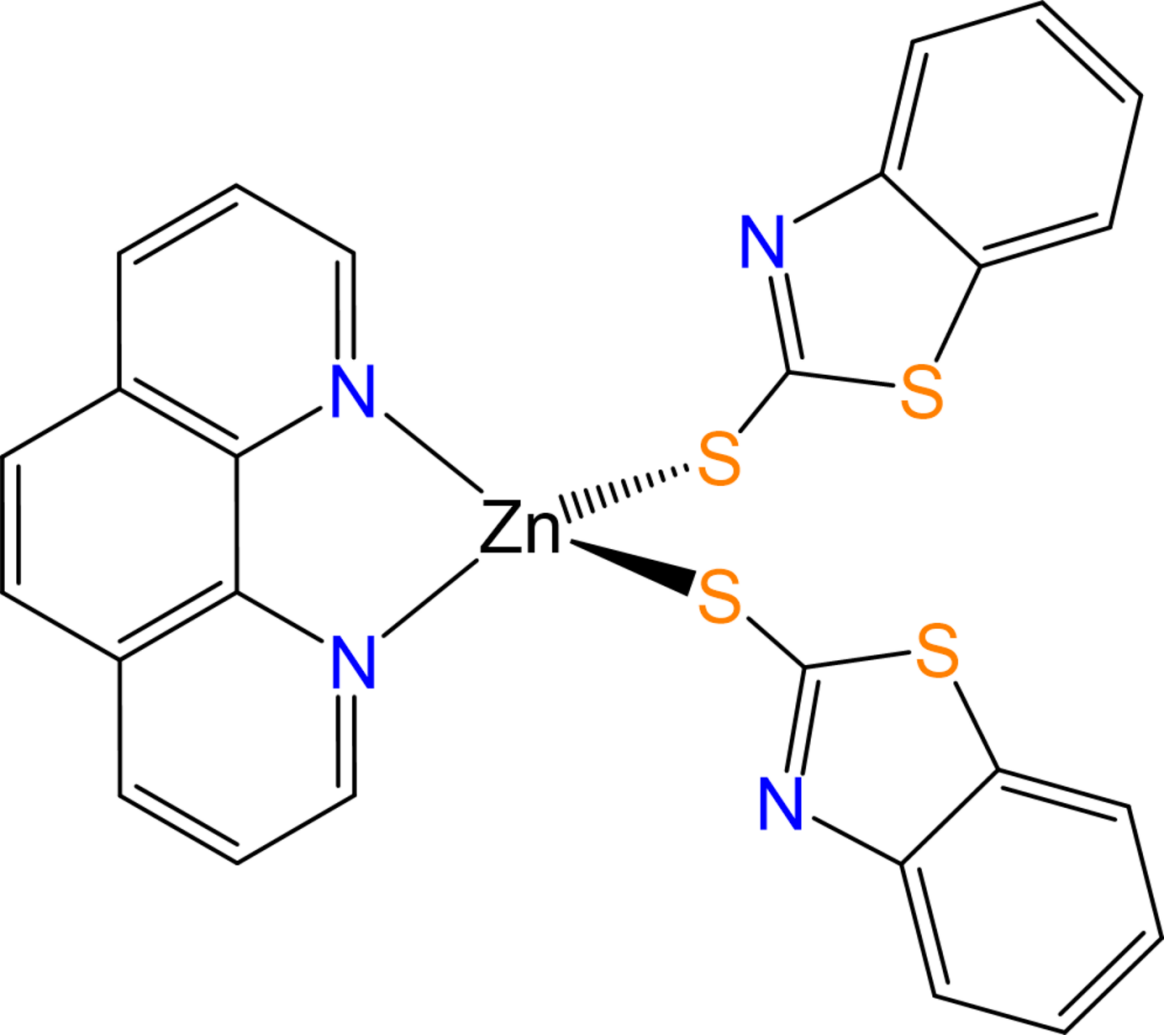


## 2. Structural commentary

**Figure 1:**
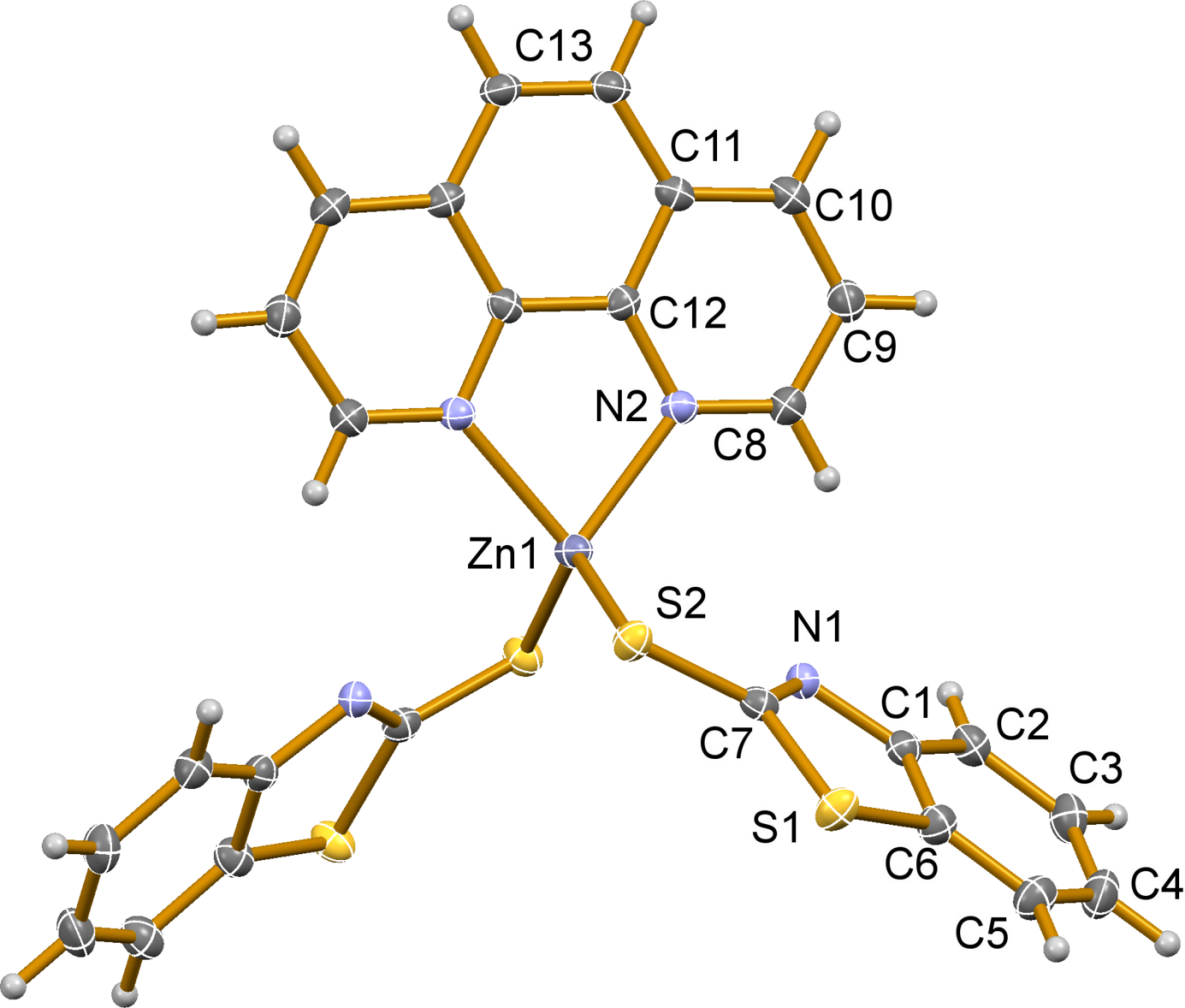
Displacement ellipsoid plot (50% ellipsoid probability level) of [Zn(MBT)_2_(phen)] showing the atom labeling. Hydrogen atoms are represented as small spheres of arbitrary radii.

[Zn(MBT)_2_(phen)] crystallizes in the monoclinic crystal system, space group *C*2/*c*. The asymmetric unit contains one 2-mercaptobenzothiazolate (MBT; anionic form of MBTH) ligand and half of a phenanthroline ligand, with the central zinc atom located on a crystallographic twofold axis (Fig. 1 ▸). The zinc atom displays a distorted tetra­hedral geometry and is chelated bidentately by a neutral phenanthroline ligand through two nitro­gen donor atoms, with a Zn—N bond length of 2.093 (2) Å. Additionally, it coordinates two MBT ligands in a monodentate fashion *via* sulfur atoms, exhibiting a Zn—S bond length of 2.2987 (7) Å. This results in a coordination number of four for the zinc center. The dihedral angle subtended by the planes through the phenanthroline ligand and the MBT ring is 52.41 (11)°.

An intra­molecular C8—H8⋯N1 contact occurs between the phenanthroline ligand and a nitro­gen atom of the MBT ligand (H⋯*A* = 2.65 Å, Table 1 ▸). This distance is consistent with reported values for weak hydrogen-bonding inter­actions, as C—H⋯N contacts are typically considered significant when the H⋯N distance is less than ∼2.75 Å and the donor (proton)–acceptor angle in a hydrogen bond must be at least 90° (Zefirov & Zorkii, 1974 ▸; Taylor & Kennard, 1982 ▸).

## 3. Supra­molecular features

**Figure 2:**
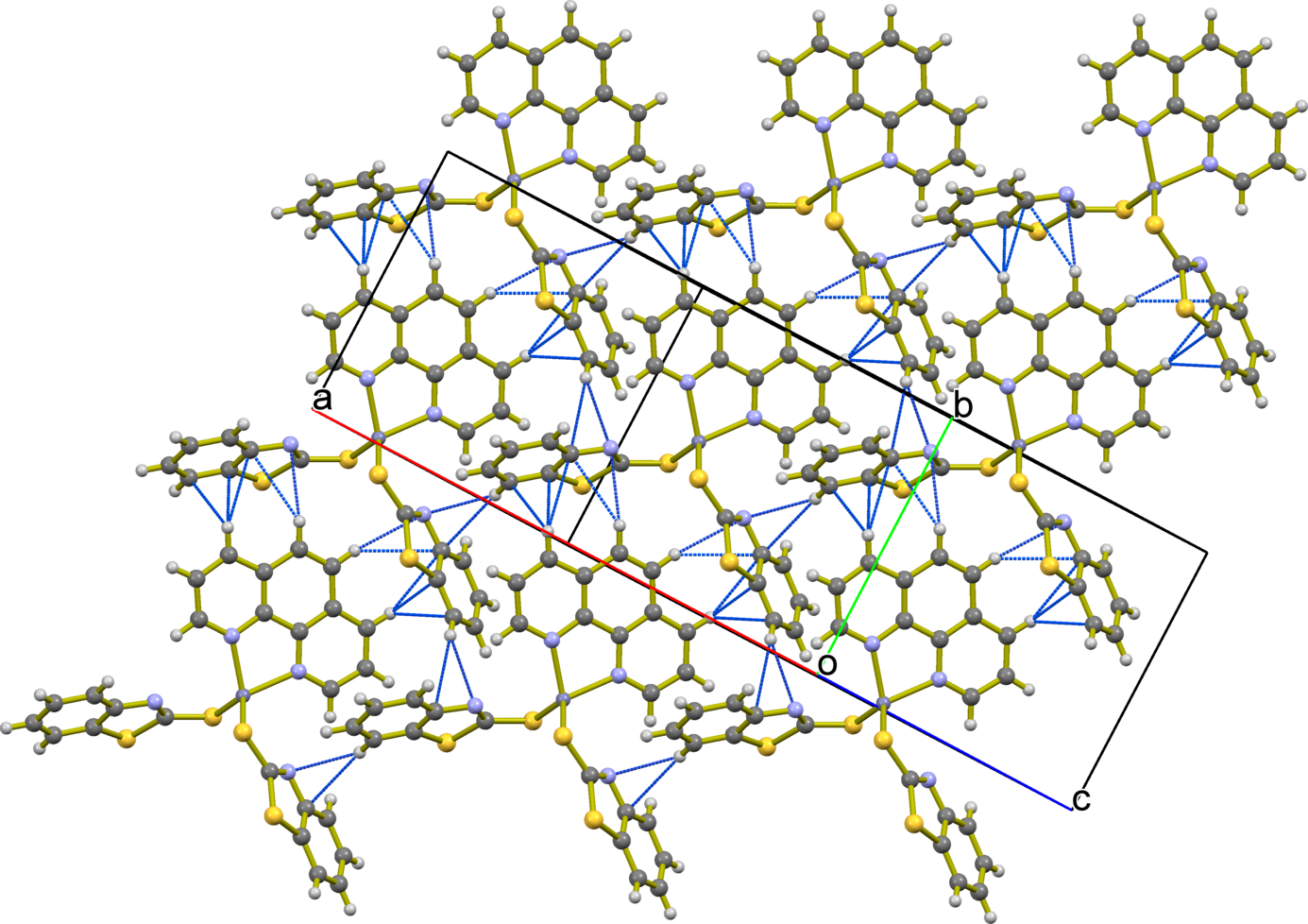
The packing of [Zn(MBT)_2_(phen)], showing the complex mol­ecules connected by C—H⋯N and C—H⋯π inter­actions.

In the crystal, several inter­molecular inter­actions are observed, including C—H⋯N and C—H⋯π inter­actions, more specifically, C5—H5⋯N1 and C13—H13⋯N1 (Table 1 ▸). The mol­ecule also exhibits several C—H⋯π inter­actions (Nishio *et al.*, 1998 ▸) including two involving the phenanthroline ring system with an MBT ring (C10—H10⋯*Cg*4^iii^ and C10—H10⋯*Cg*6^iii^; *Cg*4 and *Cg*6 are the centroids of the C1–C6 and N1/S1/C1–C7 rings, respectively; symmetry codes as in Table 1 ▸). The C—H⋯π inter­action occurs between the MBT rings of adjacent mol­ecules (C5—H5⋯*Cg*2^ii^; *Cg*2 is the centroid of the S1/C6/C1/N1/C7 ring; symmetry code as in Table 1 ▸. The packing is shown in Fig. 2 ▸.

## 4. Hirshfeld surface analysis

**Figure 3:**
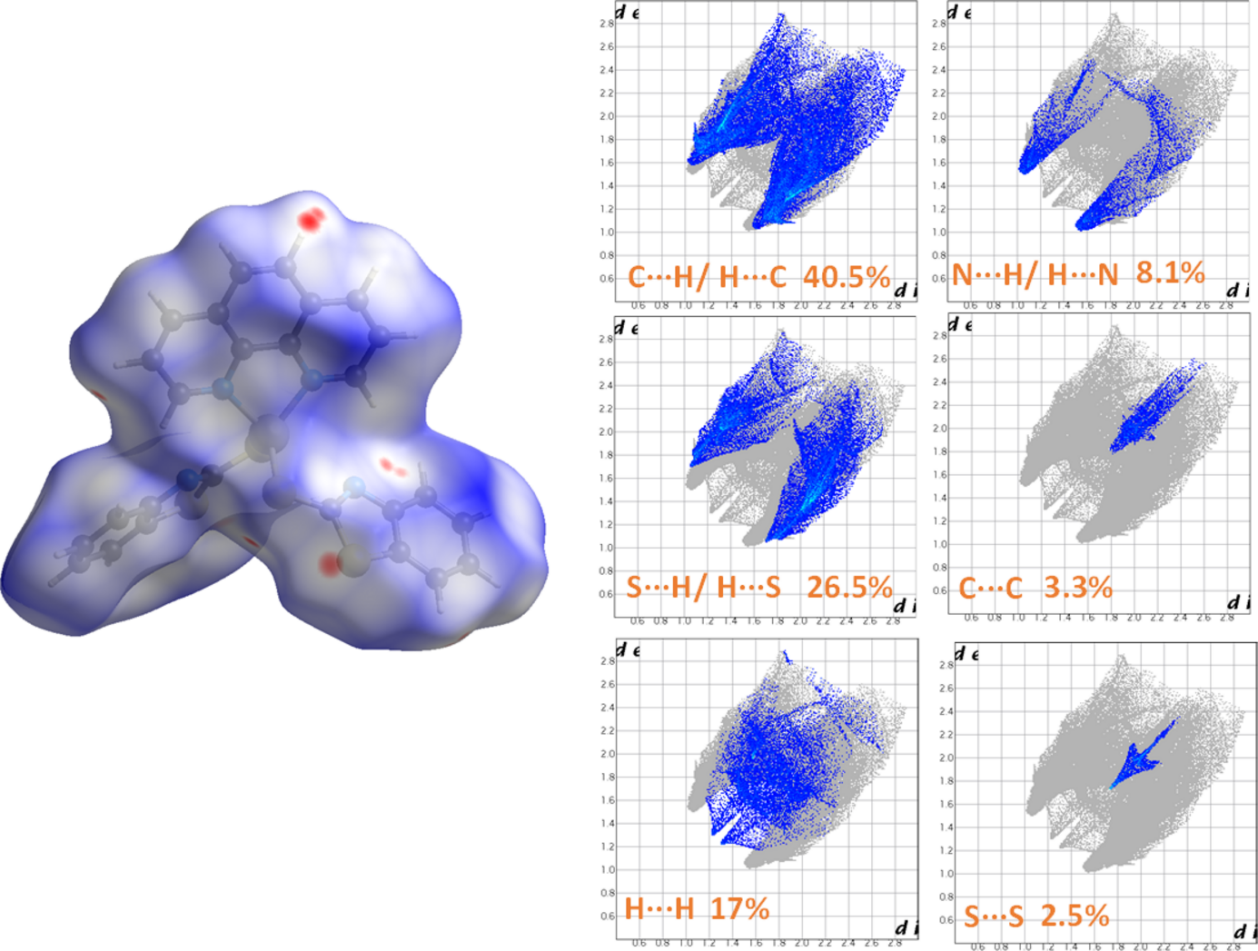
Hirshfeld surface and two-dimensional fingerprint plots for [Zn(MBT)_2_(phen)].

Hirshfeld surface analysis (Spackman & Jayatilaka, 2009 ▸) and 2D fingerprint plot analysis (Spackman & McKinnon, 2002 ▸) were carried out using *CrystalExplorer21.5* (Spackman *et al.*, 2021 ▸) to identify and qu­antify the inter­molecular inter­actions contributing to the Hirshfeld surface of the mol­ecule (Fig. 3 ▸). The structure of [Zn(MBT)_2_(phen)] primarily exhibits non-classical inter­actions including C⋯H/H⋯C, S⋯H/H⋯S, H⋯H, and N⋯H/H⋯N contacts. In addition, minor contributions from C⋯C and S⋯S inter­actions are also observed. The relative contributions of these inter­actions to the Hirshfeld surface are as follows: C⋯H/H⋯C (40.5%), S⋯H/H⋯S (26.5%), H⋯H (17.0%), N⋯H/H⋯N (8.1%), C⋯C (3.3%), and S⋯S (2.5%). Notably, two distinct red spots appear on the Hirshfeld surface, corresponding to close inter­molecular C–H⋯N contacts between adjacent mol­ecules specifically C5—H5⋯N1. Additionally, a single red spot is associated with the C9—H9⋯S2 inter­action.

## 5. Database survey

A survey conducted using ConQuest software (CSD, Version 5.46, November 2024; Groom *et al.*, 2016 ▸) within the Cambridge Structural Database revealed 284 organometallic crystal structures of MBT derivatives. Among these, 26 structures exhibit the thione tautomeric form, while the remaining 258 structures show the thiol tautomeric form. Additionally, five crystal structures containing zinc atoms coordinating mercaptobenzthia­zole have been reported (BTZTZN, Ashworth *et al.*, 1976 ▸; RIRGIJ, Jin *et al.*, 2007 ▸; TIFLED, Seo *et al.*, 2023 ▸; WEDVEG, WEDVIK, Baggio *et al.*, 1993 ▸). Among these, two structures are closely related to complex [Zn(MBT)_2_(phen)], characterized by a coordination number of four (WEDVEG, WEDVIK; Baggio *et al.*, 1993 ▸). In both structures, zinc is bonded to two MBT ligands. However, in the first structure, zinc is also bonded to two pyridine ligands monodentately, whereas in the second structure, it is bonded to one bi­pyridine ligand in a bidentate fashion. Notably, no crystal structures featuring zinc coordinating MBT and phenanthroline have been reported.

## 6. Synthesis and crystallization

Zn(CH_3_COO)_2_·2H_2_O (0.110 g, 0.5 mmol) and MBT (0.167 g, 1 mmol) were dissolved separately in ethanol (3 mL), mixed together, and stirred for 1 h at 333 K. A solution of phen (0.18 g, 1 mmol) in 3 mL of ethanol was added dropwise to the resulting mixture. The mixture was stirred for an additional 1 h at 333 K. The mixture was then filtered and left to crystallize. Single crystals of the title complex, suitable for X-ray analysis, were obtained by slow evaporation of the solution over a period of 10 days.

## 7. Refinement

Crystal data, data collection and structure refinement details are summarized in Table 2 ▸. H atoms were positioned geometrically (C—H = 0.95 Å) and refined as riding with *U*_iso_(H) = 1.2*U*_eq_(C).

## Supplementary Material

Crystal structure: contains datablock(s) I. DOI: 10.1107/S2056989025005468/ox2015sup1.cif

Structure factors: contains datablock(s) I. DOI: 10.1107/S2056989025005468/ox2015Isup2.hkl

CCDC reference: 2388092

Additional supporting information:  crystallographic information; 3D view; checkCIF report

## Figures and Tables

**Table 1:** Hydrogen-bond geometry (Å, °) *Cg*2, *Cg*4 and *Cg*6 are the centroids of the S1/C6/C1/N1/C7, C1–C6 and N1/S1/C1–C7 rings, respectively.

*D*—H⋯*A*	*D*—H	H⋯*A*	*D*⋯*A*	*D*—H⋯*A*
C8—H8⋯N1	0.95	2.65	3.331 (3)	130
C2—H2⋯S2^i^	0.95	2.98	3.745 (3)	138
C5—H5⋯N1^ii^	0.95	2.69	3.508 (4)	145
C13—H13⋯N1^iii^	0.95	2.69	3.574 (3)	155
C13—H13⋯C7^iii^	0.95	2.74	3.416 (4)	129
C9—H9⋯S2^i^	0.95	2.87	3.572 (3)	131
C5—H5⋯*Cg*2^ii^	0.95	2.93	3.674 (3)	136
C10—H10⋯*Cg*4^iii^	0.95	2.64	3.451 (3)	143
C10—H10⋯*Cg*6^iii^	0.95	2.77	3.498 (3)	135

Symmetry codes: (i) 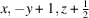; (ii) 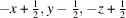; (iii) 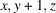.

**Table 2:** Experimental details

Crystal data	
Chemical formula	[Zn(C_7_H_4_NS_2_)_2_(C_12_H_8_N_2_)]
*M*_r_	578.04
Crystal system, space group	Monoclinic, *C*2/*c*
Temperature (K)	100
*a*, *b*, *c* (Å)	19.9559 (9), 9.8232 (5), 14.0704 (7)
β (°)	118.642 (1)
*V* (Å^3^)	2420.7 (2)
*Z*	4
Radiation type	Mo *K*α
μ (mm^−1^)	1.38
Crystal size (mm)	0.08 × 0.06 × 0.06

Data collection	
Diffractometer	Bruker APEXII CCD
Absorption correction	Multi-scan (*SADABS*; Krause et al., 2015 ▸)
*T*_min_, *T*_max_	0.525, 0.745
No. of measured, independent and observed [*I* > 2σ(*I*)] reflections	30934, 2479, 2114
*R*_int_	0.086
(sin θ/λ)_max_ (Å^−1^)	0.626

Refinement	
*R*[*F*^2^ > 2σ(*F*^2^)], *wR*(*F*^2^), *S*	0.036, 0.093, 1.08
No. of reflections	2479
No. of parameters	160
H-atom treatment	H-atom parameters constrained
Δρ_max_, Δρ_min_ (e Å^−3^)	0.53, −0.57

Computer programs: *APEX2* and *SAINT* (Bruker, 2019 ▸), *SHELXT2014/5* (Sheldrick, 2015*a* ▸), *SHELXL2016/6* (Sheldrick, 2015*b* ▸) and *OLEX2* (Dolomanov *et al.*, 2009 ▸).

## supplementary crystallographic information

### Bis(benzothiazole-2-thiolato-κS)(1,10-phenanthroline-κ2N,N')zinc(II) Crystal data

**Table:**

[Zn(C_7_H_4_NS_2_)_2_(C_12_H_8_N_2_)]	*F*(000) = 1176
*M**_r_* = 578.04	*D*_x_ = 1.586 Mg m^−^^3^
Monoclinic, *C*2/*c*	Mo *K*α radiation, λ = 0.71073 Å
*a* = 19.9559 (9) Å	Cell parameters from 9995 reflections
*b* = 9.8232 (5) Å	θ = 2.3–26.3°
*c* = 14.0704 (7) Å	µ = 1.38 mm^−^^1^
β = 118.642 (1)°	*T* = 100 K
*V* = 2420.7 (2) Å^3^	Block, colourless
*Z* = 4	0.08 × 0.06 × 0.06 mm

### Bis(benzothiazole-2-thiolato-κS)(1,10-phenanthroline-κ2N,N')zinc(II) Data collection

**Table:**

Bruker APEXII CCD diffractometer	2114 reflections with *I* > 2σ(*I*)
φ and ω scans	*R*_int_ = 0.086
Absorption correction: multi-scan (SADABS; Krause et al., 2015)	θ_max_ = 26.4°, θ_min_ = 2.6°
*T*_min_ = 0.525, *T*_max_ = 0.745	*h* = −24→24
30934 measured reflections	*k* = −12→12
2479 independent reflections	*l* = −17→17

### Bis(benzothiazole-2-thiolato-κS)(1,10-phenanthroline-κ2N,N')zinc(II) Refinement

**Table:**

Refinement on *F*^2^	Hydrogen site location: inferred from neighbouring sites
Least-squares matrix: full	H-atom parameters constrained
*R*[*F*^2^ > 2σ(*F*^2^)] = 0.036	*w* = 1/[σ^2^(*F*_o_^2^) + (0.0397*P*)^2^ + 6.4028*P*] where *P* = (*F*_o_^2^ + 2*F*_c_^2^)/3
*wR*(*F*^2^) = 0.093	(Δ/σ)_max_ = 0.001
*S* = 1.08	Δρ_max_ = 0.53 e Å^−^^3^
2479 reflections	Δρ_min_ = −0.57 e Å^−^^3^
160 parameters	Extinction correction: *SHELXL2016/6* (Sheldrick, 2015*b*), Fc^*^=kFc[1+0.001xFc^2^λ^3^/sin(2θ)]^-1/4^
0 restraints	Extinction coefficient: 0.0011 (3)

### Bis(benzothiazole-2-thiolato-κS)(1,10-phenanthroline-κ2N,N')zinc(II) Special details

**Table:**

Geometry. All esds (except the esd in the dihedral angle between two l.s. planes) are estimated using the full covariance matrix. The cell esds are taken into account individually in the estimation of esds in distances, angles and torsion angles; correlations between esds in cell parameters are only used when they are defined by crystal symmetry. An approximate (isotropic) treatment of cell esds is used for estimating esds involving l.s. planes.

### Bis(benzothiazole-2-thiolato-κS)(1,10-phenanthroline-κ2N,N')zinc(II) Fractional atomic coordinates and isotropic or equivalent isotropic displacement parameters (Å^2^)

**Table:**

	*x*	*y*	*z*	*U*_iso_*/*U*_eq_	
Zn1	0.500000	0.51950 (4)	0.250000	0.01817 (15)	
S1	0.29666 (4)	0.20222 (7)	0.13597 (6)	0.02333 (19)	
N1	0.39011 (12)	0.3587 (2)	0.29326 (18)	0.0182 (5)	
C1	0.35313 (15)	0.2772 (3)	0.3346 (2)	0.0189 (6)	
N2	0.46554 (13)	0.6818 (2)	0.31279 (18)	0.0180 (5)	
S2	0.40002 (4)	0.40663 (7)	0.11137 (5)	0.02157 (18)	
C2	0.36580 (16)	0.2815 (3)	0.4411 (2)	0.0225 (6)	
H2	0.401484	0.343781	0.491642	0.027*	
C3	0.32539 (17)	0.1932 (3)	0.4717 (2)	0.0268 (6)	
H3	0.333635	0.195473	0.544039	0.032*	
C4	0.27273 (17)	0.1008 (3)	0.3983 (3)	0.0294 (7)	
H4	0.245972	0.040938	0.421358	0.035*	
C5	0.25921 (17)	0.0956 (3)	0.2922 (2)	0.0263 (6)	
H5	0.223334	0.033212	0.242063	0.032*	
C6	0.29951 (16)	0.1843 (3)	0.2609 (2)	0.0216 (6)	
C7	0.36666 (15)	0.3307 (3)	0.1914 (2)	0.0184 (5)	
C10	0.43160 (16)	0.9233 (3)	0.3852 (2)	0.0214 (6)	
H10	0.420249	1.004882	0.410930	0.026*	
C11	0.46615 (15)	0.9288 (3)	0.3182 (2)	0.0184 (5)	
C12	0.48230 (15)	0.8048 (3)	0.2842 (2)	0.0168 (5)	
C13	0.48445 (16)	1.0540 (3)	0.2834 (2)	0.0215 (6)	
H13	0.474609	1.138154	0.307709	0.026*	
C9	0.41457 (16)	0.7996 (3)	0.4129 (2)	0.0234 (6)	
H9	0.390538	0.794423	0.456981	0.028*	
C8	0.43297 (16)	0.6802 (3)	0.3755 (2)	0.0213 (6)	
H8	0.421575	0.594830	0.396097	0.026*	

### Bis(benzothiazole-2-thiolato-κS)(1,10-phenanthroline-κ2N,N')zinc(II) Atomic displacement parameters (Å^2^)

**Table:**

	*U*^11^	*U*^22^	*U*^33^	*U*^12^	*U*^13^	*U*^23^
Zn1	0.0208 (3)	0.0147 (2)	0.0191 (3)	0.000	0.00969 (19)	0.000
S1	0.0229 (4)	0.0248 (4)	0.0183 (4)	−0.0073 (3)	0.0067 (3)	−0.0024 (3)
N1	0.0168 (11)	0.0182 (11)	0.0205 (11)	0.0002 (9)	0.0097 (9)	−0.0001 (9)
C1	0.0156 (13)	0.0179 (13)	0.0227 (14)	0.0018 (11)	0.0088 (11)	0.0004 (11)
N2	0.0190 (11)	0.0157 (10)	0.0193 (11)	−0.0006 (9)	0.0091 (9)	−0.0010 (9)
S2	0.0231 (4)	0.0227 (3)	0.0192 (4)	−0.0037 (3)	0.0104 (3)	−0.0009 (3)
C2	0.0206 (14)	0.0247 (14)	0.0222 (14)	0.0015 (11)	0.0102 (12)	−0.0025 (11)
C3	0.0263 (16)	0.0347 (16)	0.0240 (15)	0.0017 (13)	0.0158 (13)	0.0015 (12)
C4	0.0263 (16)	0.0333 (16)	0.0344 (17)	−0.0019 (13)	0.0191 (14)	0.0050 (13)
C5	0.0207 (15)	0.0267 (15)	0.0282 (16)	−0.0039 (12)	0.0092 (12)	0.0014 (12)
C6	0.0178 (14)	0.0226 (14)	0.0221 (14)	−0.0016 (11)	0.0076 (11)	0.0003 (11)
C7	0.0171 (13)	0.0164 (12)	0.0191 (13)	0.0028 (10)	0.0065 (11)	−0.0002 (10)
C10	0.0208 (14)	0.0207 (13)	0.0210 (14)	0.0032 (11)	0.0087 (12)	−0.0022 (11)
C11	0.0173 (13)	0.0176 (13)	0.0166 (13)	0.0016 (10)	0.0052 (11)	−0.0009 (10)
C12	0.0127 (12)	0.0176 (12)	0.0171 (13)	−0.0009 (10)	0.0048 (10)	0.0011 (10)
C13	0.0209 (14)	0.0149 (12)	0.0272 (15)	0.0009 (10)	0.0104 (12)	−0.0011 (11)
C9	0.0226 (15)	0.0252 (14)	0.0247 (15)	0.0001 (11)	0.0132 (12)	−0.0013 (12)
C8	0.0211 (14)	0.0204 (13)	0.0239 (14)	−0.0011 (11)	0.0119 (12)	0.0000 (11)

### Bis(benzothiazole-2-thiolato-κS)(1,10-phenanthroline-κ2N,N')zinc(II) Geometric parameters (Å, º)

**Table:**

Zn1—N2	2.093 (2)	C3—C4	1.398 (4)
Zn1—N2^i^	2.093 (2)	C4—H4	0.9500
Zn1—S2^i^	2.2987 (7)	C4—C5	1.384 (4)
Zn1—S2	2.2987 (7)	C5—H5	0.9500
S1—C6	1.740 (3)	C5—C6	1.393 (4)
S1—C7	1.763 (3)	C10—H10	0.9500
N1—C1	1.392 (3)	C10—C11	1.411 (4)
N1—C7	1.306 (4)	C10—C9	1.368 (4)
C1—C2	1.395 (4)	C11—C12	1.402 (4)
C1—C6	1.410 (4)	C11—C13	1.434 (4)
N2—C12	1.364 (3)	C12—C12^i^	1.442 (5)
N2—C8	1.324 (4)	C13—C13^i^	1.353 (6)
S2—C7	1.728 (3)	C13—H13	0.9500
C2—H2	0.9500	C9—H9	0.9500
C2—C3	1.387 (4)	C9—C8	1.404 (4)
C3—H3	0.9500	C8—H8	0.9500
			
N2—Zn1—N2^i^	80.71 (12)	C6—C5—H5	120.8
N2—Zn1—S2^i^	109.67 (6)	C1—C6—S1	108.9 (2)
N2^i^—Zn1—S2^i^	113.49 (6)	C5—C6—S1	129.7 (2)
N2—Zn1—S2	113.49 (6)	C5—C6—C1	121.4 (3)
N2^i^—Zn1—S2	109.66 (6)	N1—C7—S1	115.3 (2)
S2—Zn1—S2^i^	122.32 (4)	N1—C7—S2	125.2 (2)
C6—S1—C7	89.44 (13)	S2—C7—S1	119.47 (15)
C7—N1—C1	110.8 (2)	C11—C10—H10	120.3
N1—C1—C2	124.9 (2)	C9—C10—H10	120.3
N1—C1—C6	115.6 (2)	C9—C10—C11	119.5 (3)
C2—C1—C6	119.5 (3)	C10—C11—C13	123.2 (2)
C12—N2—Zn1	111.95 (17)	C12—C11—C10	117.4 (2)
C8—N2—Zn1	129.67 (18)	C12—C11—C13	119.4 (2)
C8—N2—C12	118.4 (2)	N2—C12—C11	122.7 (2)
C7—S2—Zn1	95.97 (9)	N2—C12—C12^i^	117.68 (15)
C1—C2—H2	120.6	C11—C12—C12^i^	119.63 (15)
C3—C2—C1	118.8 (3)	C11—C13—H13	119.5
C3—C2—H2	120.6	C13^i^—C13—C11	120.94 (16)
C2—C3—H3	119.4	C13^i^—C13—H13	119.5
C2—C3—C4	121.3 (3)	C10—C9—H9	120.3
C4—C3—H3	119.4	C10—C9—C8	119.4 (3)
C3—C4—H4	119.7	C8—C9—H9	120.3
C5—C4—C3	120.6 (3)	N2—C8—C9	122.7 (3)
C5—C4—H4	119.7	N2—C8—H8	118.6
C4—C5—H5	120.8	C9—C8—H8	118.6
C4—C5—C6	118.3 (3)		
			
Zn1—N2—C12—C11	−178.7 (2)	C6—C1—C2—C3	0.4 (4)
Zn1—N2—C12—C12^i^	1.6 (4)	C7—S1—C6—C1	0.1 (2)
Zn1—N2—C8—C9	178.7 (2)	C7—S1—C6—C5	−179.0 (3)
Zn1—S2—C7—S1	−169.30 (14)	C7—N1—C1—C2	179.5 (3)
Zn1—S2—C7—N1	9.4 (2)	C7—N1—C1—C6	−0.2 (3)
N1—C1—C2—C3	−179.4 (3)	C10—C11—C12—N2	0.4 (4)
N1—C1—C6—S1	0.0 (3)	C10—C11—C12—C12^i^	−179.9 (3)
N1—C1—C6—C5	179.2 (3)	C10—C11—C13—C13^i^	−178.0 (3)
C1—N1—C7—S1	0.3 (3)	C10—C9—C8—N2	−0.9 (4)
C1—N1—C7—S2	−178.5 (2)	C11—C10—C9—C8	1.0 (4)
C1—C2—C3—C4	0.1 (4)	C12—N2—C8—C9	0.5 (4)
C2—C1—C6—S1	−179.7 (2)	C12—C11—C13—C13^i^	1.4 (5)
C2—C1—C6—C5	−0.5 (4)	C13—C11—C12—N2	−179.1 (3)
C2—C3—C4—C5	−0.4 (5)	C13—C11—C12—C12^i^	0.6 (4)
C3—C4—C5—C6	0.2 (4)	C9—C10—C11—C12	−0.8 (4)
C4—C5—C6—S1	179.2 (2)	C9—C10—C11—C13	178.7 (3)
C4—C5—C6—C1	0.2 (4)	C8—N2—C12—C11	−0.3 (4)
C6—S1—C7—N1	−0.2 (2)	C8—N2—C12—C12^i^	−179.9 (3)
C6—S1—C7—S2	178.61 (17)		

Symmetry code: (i) −*x*+1, *y*, −*z*+1/2.

### Bis(benzothiazole-2-thiolato-κS)(1,10-phenanthroline-κ2N,N')zinc(II) Hydrogen-bond geometry (Å, º)

*Cg*2, *Cg*4 and *Cg*6 are the centroids of the S1/C6/C1/N1/C7, C1–C6 and N1/S1/C1–C6 rings, respectively.

**Table:**

*D*—H···*A*	*D*—H	H···*A*	*D*···*A*	*D*—H···*A*
C8—H8···N1	0.95	2.65	3.331 (3)	130
C2—H2···S2^ii^	0.95	2.98	3.745 (3)	138
C5—H5···N1^iii^	0.95	2.69	3.508 (4)	145
C13—H13···N1^iv^	0.95	2.69	3.574 (3)	155
C13—H13···C7^iv^	0.95	2.74	3.416 (4)	129
C9—H9···S2^ii^	0.95	2.87	3.572 (3)	131
C5—H5···*Cg*2^iii^	0.95	2.93	3.674 (3)	136
C10—H10···*Cg*4^iv^	0.95	2.64	3.451 (3)	143
C10—H10···*Cg*6^iv^	0.95	2.77	3.498 (3)	135

Symmetry codes: (ii) *x*, −*y*+1, *z*+1/2; (iii) −*x*+1/2, *y*−1/2, −*z*+1/2; (iv) *x*, *y*+1, *z*.
